# Diagnostic Value of the Split-Bolus CT Protocol in Blunt Abdominal Trauma: Comparison With Standard Portal Venous Phase Imaging

**DOI:** 10.7759/cureus.93563

**Published:** 2025-09-30

**Authors:** Arpit Taneja, Narvir S Chauhan, Pankaj Saini, Dinesh Sood, Aman Taneja

**Affiliations:** 1 Radiology, Dr. Rajendra Prasad Government Medical College, Tanda, IND; 2 Radiology, Teerthanker Mahaveer Medical College and Research Center, Moradabad, IND

**Keywords:** blunt abdominal trauma, diagnostic imaging, portal venous phase, solid organ injury, split-bolus ct

## Abstract

Background

Blunt abdominal trauma is a significant cause of morbidity and mortality, particularly in young adults, and accurate imaging is essential for timely diagnosis and management. Computed tomography (CT) is the preferred modality in hemodynamically stable patients, with the routine portal venous (PV) phase protocol commonly employed. Recently, the split-bolus (SB) protocol has been proposed as an alternative, enabling simultaneous arterial and venous phase visualization in a single acquisition. This study compared the image quality, contrast enhancement, diagnostic accuracy, and radiation exposure of the SB protocol versus the standard PV protocol in evaluating blunt abdominal trauma.

Methods

A prospective observational study was conducted from September 2021 to August 2022 at Dr. Rajendra Prasad Government Medical College, Kangra. Seventy hemodynamically stable patients with suspected intra-abdominal injury were assigned to SB (n = 35) or PV (n = 35) CT protocols using a non-probability convenience sampling method. Organ and vascular attenuation values were measured in Hounsfield units (HU). Image quality was assessed on a five-point Likert scale, and organ injuries were graded using the Organ Injury Scale (OIS) by two experienced radiologists. Radiation dose metrics included the volume CT dose index (CTDIvol, mGy) and dose-length product (DLP, mGy·cm), which were recorded directly from the scanner console. Effective dose (mSv) was calculated by multiplying DLP by the standard abdominal conversion factor (k = 0.015). Statistical analysis included t-tests, chi-square tests, and weighted kappa statistics for inter-reader agreement, with p < 0.05 considered significant.

Results

The study population had a mean age of 35.6 ± 13.7 years, with a male predominance (75.7%). Solid organ injuries were identified in 88.6% of cases, most frequently involving the liver (52.9%) and spleen (34.3%). The SB protocol demonstrated significantly higher enhancement in the spleen (141.7 ± 27.8 HU vs. 119.6 ± 19.8 HU, p < 0.001), pancreas (121.7 ± 22.1 HU vs. 99.9 ± 13.6 HU, p < 0.001), and renal cortex (240.6 ± 45.1 HU vs. 179.9 ± 31.4 HU, p < 0.001), while hepatic enhancement was comparable (p = 0.834). Vascular enhancement was consistently superior with SB, particularly in the portal vein, aorta, inferior vena cava, and iliac vessels (all p < 0.05). Overall image quality was rated as good to excellent in both protocols, with no significant difference. Importantly, SB facilitated simultaneous evaluation of arterial and venous injuries and improved urinary tract opacification, reducing the need for delayed scans. Inter-reader agreement for OIS scoring was moderate to substantial (κ = 0.563 for SB; κ = 0.639 for PV). The mean CTDIvol and DLP were comparable between SB and PV groups (CTDIvol: X vs. Y mGy; DLP: X vs. Y mGy·cm, both p > 0.05). The derived effective dose was also similar (8.87 ± 2.85 mSv vs. 8.89 ± 3.05 mSv, p = 0.846).

Conclusions

The SB CT protocol provided superior vascular and parenchymal enhancement compared to the routine PV protocol without increasing radiation exposure. By consolidating arterial and venous phases into a single scan, SB streamlined trauma evaluation, enhanced diagnostic confidence in vascular and urinary tract injuries, and reduced the need for multiphase acquisitions.

## Introduction

Trauma is one of the foremost public health challenges worldwide and remains the leading cause of death in individuals younger than 45 years [1]. Among these cases, abdominal injuries contribute to nearly 10% of trauma-related fatalities, often posing diagnostic and therapeutic challenges [2]. Road traffic accidents (RTAs) are the second most common cause of mortality among individuals aged five to 29 years [3]. In India, the burden is particularly high in the 15-25-year age group, which experiences disproportionate exposure to high-risk activities such as two-wheeler driving and occupational hazards. Developing countries such as India and China have witnessed a rapid rise in RTA-related deaths, whereas high-income nations have experienced a slower, more controlled increase due to stricter traffic regulations, better trauma systems, and improved emergency care infrastructure [1]. Since approximately 30% of these trauma-related deaths are considered preventable, prompt diagnosis and timely intervention are crucial in lowering morbidity and mortality [4].

Blunt abdominal trauma, in particular, can be difficult to diagnose, as external signs of injury are often absent or misleading, allowing internal injuries to go undetected until they become life-threatening. Early and accurate diagnosis is, therefore, of paramount importance. Among the diagnostic modalities available, computed tomography (CT) has emerged as the gold standard for evaluating hemodynamically stable patients [5,6]. CT offers rapid, high-resolution visualization of intra-abdominal organs, the retroperitoneum, abdominal wall, and associated structures [7]. Beyond abdominal assessment, CT also plays a vital role in detecting concurrent injuries, such as thoracic trauma, pelvic fractures, and spinal injuries, which often coexist in polytrauma patients. Furthermore, its ability to produce high-quality multiplanar reconstructions in a short time significantly enhances diagnostic precision and aids in surgical planning [8].

Over the years, the role of CT in blunt abdominal trauma has continued to expand. Multidetector CT (MDCT), in particular, has proven highly effective in detecting solid organ injuries, active hemorrhage, bowel and mesenteric trauma, and vascular injuries, with excellent sensitivity and specificity [9]. The introduction of contrast-enhanced CT has revolutionized trauma imaging, reducing the reliance on invasive procedures such as diagnostic peritoneal lavage. By providing accurate anatomical and functional information, CT has firmly established itself as an indispensable tool in trauma management [10].

Another important impact of CT imaging is its influence on the management of solid organ injuries. Historically, many abdominal trauma cases were treated operatively due to diagnostic uncertainty. However, with advances in CT, the trend has shifted toward selective nonoperative management, especially for liver and splenic injuries [11,12]. While surgical decisions continue to be guided primarily by clinical findings, CT increases diagnostic confidence and helps avoid unnecessary exploratory laparotomies [12].

To further optimize trauma imaging, innovative CT protocols have been developed. One such advancement is the split-bolus (SB) technique, which aims to combine the diagnostic advantages of multi-phase imaging while minimizing radiation exposure. Traditional trauma CT often requires multiple acquisitions at different phases of contrast enhancement (arterial, portal venous, and delayed), resulting in increased radiation dose and a more complex imaging workflow, even though the actual gantry scan time remains unchanged. In contrast, the SB protocol merges arterial and venous phases into a single acquisition by dividing the contrast medium into multiple boluses administered at timed intervals. This not only reduces the number of acquisitions and radiation dose but also provides a comprehensive vascular and parenchymal assessment in a streamlined manner [13].

In many institutions, a serial two-phase CT protocol is commonly used, with both arterial and portal venous phases obtained from separate acquisitions after a single contrast injection. The SB technique offers an alternative approach, enabling simultaneous visualization of both phases in a single acquisition while potentially reducing radiation dose. Given the growing emphasis on radiation safety, cost-effectiveness, and rapid workflow in trauma care, evaluating the efficacy of SB CT protocols has become highly relevant. Although prior studies have explored SB CT in polytrauma and pediatric settings, there is a paucity of evidence directly comparing the triple-split-bolus protocol with the conventional portal venous phase protocol in adult blunt abdominal trauma. This study specifically aimed to address this gap by comparing image quality, diagnostic accuracy, and radiation exposure between these two protocols. By doing so, it aims to determine whether the SB technique can serve as a reliable and efficient alternative to routine imaging strategies in trauma evaluation.

## Materials and methods

Study design

This prospective observational study was conducted in the Department of Radiodiagnosis at Dr. Rajendra Prasad Government Medical College, Kangra, Tanda, over a period of one year, from September 1, 2021, to August 31, 2022. The study was initiated after obtaining approval from the Institutional Ethics Committee (IEC/56/2021; dated 28/08/2021). Patients presenting to the emergency department with a history of blunt abdominal trauma were consecutively enrolled and randomly assigned to undergo CT imaging using either the SB protocol or the routine portal venous (PV) protocol. Enrollment was carried out in accordance with predefined inclusion and exclusion criteria to ensure uniformity and reliability in data collection. Written informed consent was obtained from all participants prior to inclusion in the study.

Sample size

The sample size was calculated using the formula, n = ([Zα/2 × P(1−P)]/d²), where Zα/2 (1.96) corresponds to a 95% confidence interval, P (0.07) represents the expected proportion, and d (0.07) denotes the precision. Based on this calculation, the estimated minimum sample size was approximately 100 patients. However, due to study limitations, a total of 70 patients who met the inclusion criteria were ultimately enrolled, with 35 patients assigned to the SB cohort and 35 to the PV cohort. The reduced sample size was primarily attributable to a 20% attrition rate, largely influenced by restrictions and operational challenges during the COVID-19 pandemic. Additionally, 10 patients were excluded because of technical issues, such as contrast extravasation and image distortion related to motion or breathing artifacts. These artifacts precluded reliable measurement of attenuation values and injury grading, which were the primary study endpoints. Participant selection was carried out using a non-probability convenience sampling method; therefore, true randomization was not performed.

Inclusion and exclusion criteria

The study population comprised patients with blunt abdominal trauma who were either positive on FAST (Focused Assessment with Sonography for Trauma) or negative on FAST but presented with a strong clinical suspicion of intra-abdominal injury, provided they were hemodynamically stable at the time of evaluation. A small subset (n = 6) was included on the basis of clinical suspicion despite a negative FAST; excluding these patients in a sensitivity analysis did not alter the study conclusions. Strong clinical suspicion was defined by the presence of abdominal tenderness, abdominal distension, unexplained hemodynamic changes (tachycardia or hypotension), or high-risk mechanisms of injury such as high-velocity RTA or a fall from height. Patients were excluded if they were hemodynamically unstable, pregnant, had impaired renal function, sustained trauma unrelated to the abdomen (such as penetrating injuries), or declined to provide informed consent.

Multi-detector computed tomography

The study was performed using a 128-slice Philips MDCT scanner (Incisive CT, Philips, Amsterdam, Netherlands). Imaging parameters included a collimation of 64 × 0.625 mm, tube voltage of 120 kV, slice thickness of 1.5 mm, increment of 0.75, filter B, and a matrix size of 512 × 512. The field of view was dynamically adjusted to ensure optimal anatomical coverage while minimizing unnecessary radiation exposure. Contrast medium was delivered via a dual-head Apollo APO 200 pressure injector (Apollo RT, Kowloon, Hong Kong). Patients were scanned in the supine position, feet first, with arms raised above the head to reduce motion artifacts and improve image quality. All patients underwent a non-contrast helical CT scan of the abdomen, extending from the diaphragmatic domes to the pubic symphysis. In the SB protocol, 150 mL of contrast was administered in three sequential phases: 20 mL for urinary tract opacification at a rate of 3 mL/s, followed by 80 mL for PV enhancement at 3 mL/s after 300 seconds, and finally 50 mL for arterial enhancement at 3 mL/s after 30 seconds, with image acquisition initiated 30 seconds after this final bolus. In the routine PV protocol, a single bolus injection of 150 mL contrast was administered at 3 mL/s, with image acquisition performed after an 80-second delay. Although the total contrast volume (150 mL) is higher than many contemporary trauma CT protocols (typically 80-100 mL), this volume was selected to achieve adequate enhancement across both vascular and parenchymal structures within the SB design. This difference is acknowledged as a study limitation (Table 1).

**Table 1:** Contrast administration protocols for split-bolus (SB) and portal venous (PV) CT in blunt abdominal trauma.

Protocol	Contrast volume	Rate	Timing	Acquisition
Split-bolus (SB)	20 mL (urography) + 80 mL (PV) + 50 mL (arterial)	3 mL/s	300 s + 30 s	Single acquisition after final bolus
Portal venous (PV)	150 mL	3 mL/s	80 s	Single acquisition

Image evaluation

A single radiologist evaluated the radiodensity of key abdominal organs and vascular structures. Attenuation values, expressed in Hounsfield units (HU), were obtained for the liver, spleen, pancreas, right renal cortex, renal pelvis, adrenal glands, and psoas muscle by placing regions of interest (ROI) measuring 2-3 cm² within non-injured parenchymal areas, while carefully avoiding inclusion of major vessels. For vascular assessment, including the abdominal aorta, inferior vena cava (IVC), portal vein, right common iliac artery, and right common iliac vein, attenuation was measured using ROIs of 0.5-1 cm², positioned strictly within the vessel lumen without including the vessel wall. This standardized approach ensured uniform, consistent, and accurate radiodensity measurements across all study participants.

Radiation dose calculation

The study employed an automatic exposure control (AEC) system to optimize radiation dose according to patient size and body habitus. Radiation dose optimization was performed using the DoseRight Automatic Exposure Control system (Philips Healthcare, Amsterdam, Netherlands), which incorporates three key components: (a) automatic current selection; (b) D-DOM for angular dose modulation; and (c) Z-DOM for longitudinal dose modulation. Detailed descriptions of the system and its functioning have been published previously [14]. These features work synergistically to preserve diagnostic image quality while minimizing unnecessary radiation exposure. The system operates on a reference image concept, wherein the operator selects a protocol-specific milliampere-second (mAs) value. Using the attenuation information from the scout projection radiograph (SPR), the system automatically modulates exposure to maintain a consistent noise level across varying patient anatomies. Radiation dose metrics included the volume CT dose index (CTDIvol, mGy) and dose-length product (DLP, mGy·cm), which were recorded directly from the scanner console. Effective dose (mSv) was calculated by multiplying DLP by the standard abdominal conversion factor (k = 0.015).

Evaluation of organ injury

Image quality was evaluated using the Organ Injury Scale (OIS), a standardized five-point Likert scale, where scores were defined as follows: 1 = non-diagnostic, 2 = poor, 3 = moderate, 4 = good, and 5 = excellent [15]. Organ injuries were graded according to the OIS by two independent radiologists, each with more than 10 years of experience in abdominal trauma imaging, who were blinded to the CT protocol (SB vs. PV) to minimize bias. To minimize bias, the assessments were performed independently and at different time intervals. All CT datasets were reviewed at a dedicated workstation using a standardized display protocol. Axial, coronal, and sagittal reconstructions, along with maximum intensity projection (MIP)/multiplanar reformation (MPR)/volume rendering (VR) series, were generated automatically before review. Radiologists interpreted these pre-prepared datasets, ensuring uniformity and minimizing variability. Since both SB and PV protocols resulted in a single acquisition dataset at the PV time frame, the readers were effectively blinded to the protocol type during OIS grading.

Statistical analysis

Data were analyzed using Microsoft Excel (Microsoft Corporation, Redmond, WA) and SPSS version 23.0 (IBM Corp., Armonk, NY). Continuous variables were summarized as mean ± standard deviation (SD), and categorical variables as frequencies and percentages. Normality was assessed with the Shapiro-Wilk test. Between-group comparisons used the Student’s t-test or Wilcoxon-Mann-Whitney U-test, as appropriate. Categorical data were analyzed with the chi-square or Fisher’s exact test. Inter-reader agreement for organ injury grading was assessed using weighted kappa statistics. A p-value <0.05 was considered statistically significant.

## Results

A total of 70 patients were included in the study, with a male predominance of 53 (75.7%). The mean age was 35.6 ± 13.7 years (median = 33.5 years, IQR = 25.8-42.8), ranging from nine to 70 years. The most common age group was 31-40 years with 22 (31.4%) patients, followed by 21-30 years with 16 (22.9%), 41-50 years with 12 (17.1%), and ≤20 years with 10 (14.3%) patients, while 10 (14.2%) patients were older than 50 years. Intra-abdominal solid organ injuries were identified in 62 (88.6%) cases. The liver was the most frequently injured organ in 37 (52.9%) patients, followed by the spleen in 24 (34.3%), adrenal glands in 11 (15.7%), and kidneys in seven (10%), while pancreatic injury was rare and seen in one (1.4%) patient. The relatively high rate of solid organ injuries (88.6%) in our cohort is attributable to the inclusion criteria, which mandated either a positive FAST or a strong clinical suspicion of intra-abdominal trauma in hemodynamically stable patients. This selection bias enriched the study population with patients more likely to have intra-abdominal injuries (Table 2).

**Table 2:** Baseline demographic and clinical characteristics of the study cohort (n = 70).

Variable	Domain	Value
Age, years (mean ± SD)		35.6 ± 13.7
Age, median (IQR)		33.5 (25.8–42.8)
Age group (n, %)	≤20 years	10 (14.3%)
	21–30 years	16 (22.9%)
	31–40 years	22 (31.4%)
	41–50 years	12 (17.1%)
	>50 years	10 (14.3%)
Gender (n, %)	Male	53 (75.7%)
	Female	17 (24.3%)
Organ injured (n, %)	Liver	37 (52.9%)
	Spleen	24 (34.3%)
	Adrenal glands	11 (15.7%)
	Kidneys	7 (10.0%)
	Pancreas	1 (1.4%)

**Figure 1:**
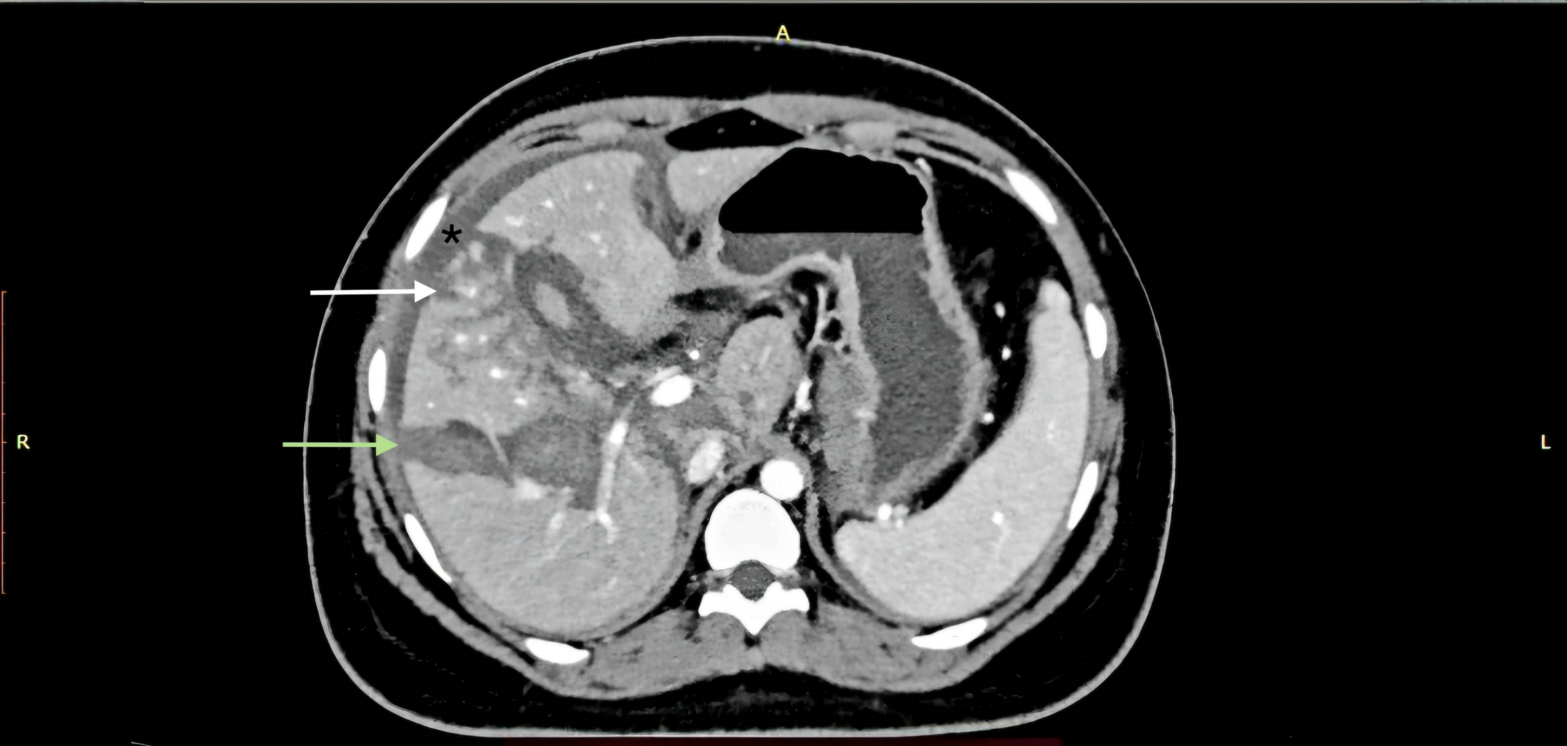
AAST grade IV liver injury showing parenchymal laceration with subcapsular hematoma on CECT (SB protocol). A 19-year-old female with a history of RTA underwent CECT of the abdomen using the SB protocol. The axial CECT image shows a large hepatic parenchymal laceration involving segments V (white arrow) and VIII (green arrow), as well as a subcapsular hematoma (black star), without active contrast extravasation. Findings were consistent with the AAST grade IV liver injury. AAST: American Association for the Surgery of Trauma; CECT: contrast-enhanced computed tomography; SB: split-bolus; RTA: road traffic accident.

**Figure 2:**
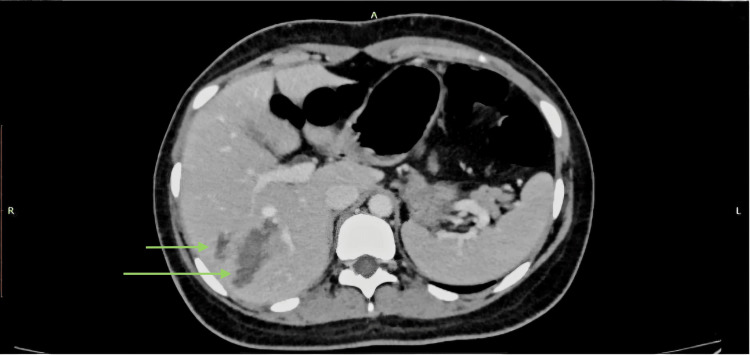
AAST grade II liver injury with linear intraparenchymal lacerations on CECT (PV protocol). A 20-year-old female with a history of RTA underwent CECT of the abdomen using the PV phase. The axial CECT image shows two linear intraparenchymal lacerations involving segments VI and VIII (green arrows) without active contrast extravasation. Findings were consistent with the AAST grade II liver injury. AAST: American Association for the Surgery of Trauma; CECT: contrast-enhanced computed tomography; PV: portal venous; RTA: road traffic accident.

**Figure 3:**
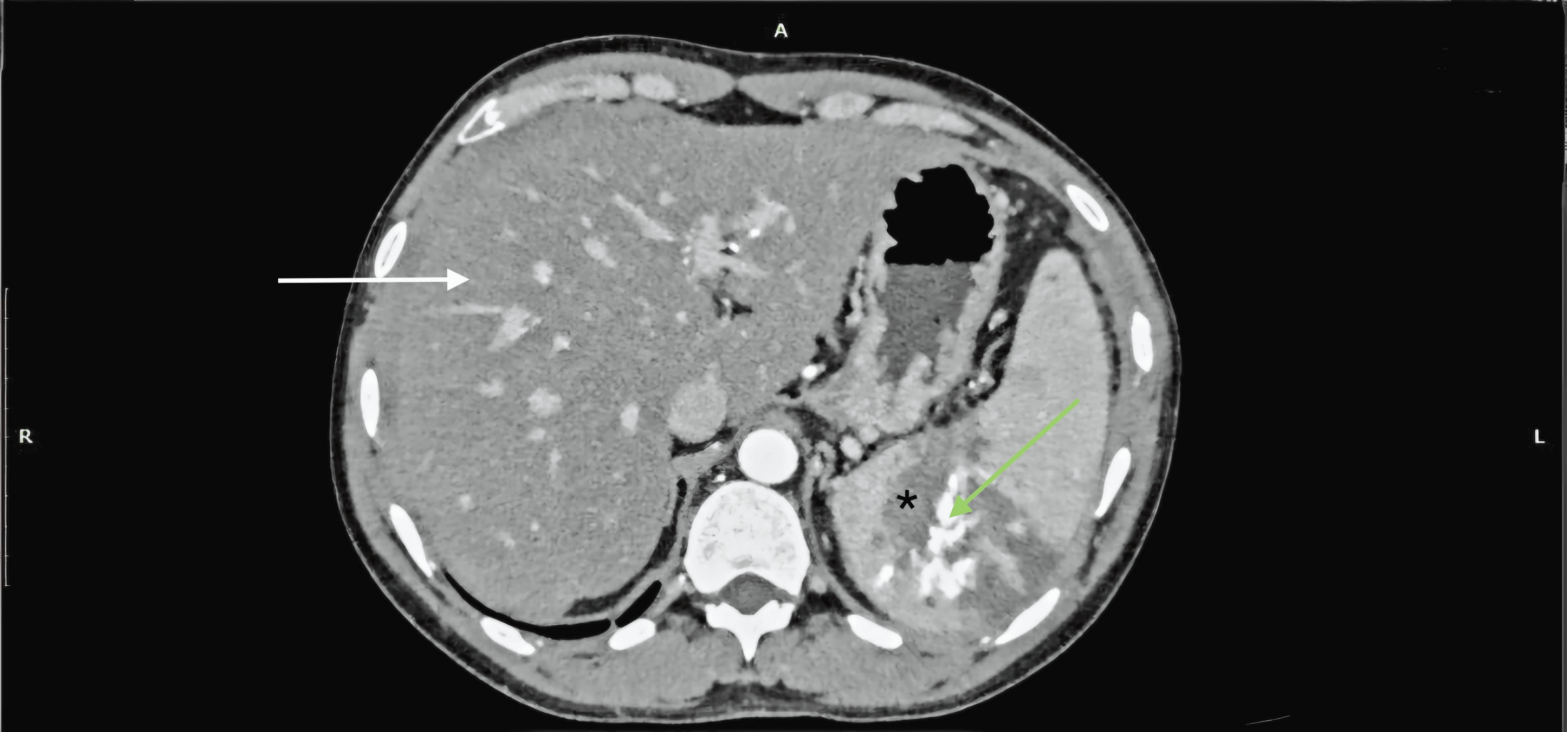
AAST grade IV splenic injury with parenchymal laceration and active contrast extravasation on CECT (SB protocol). A 33-year-old male with a history of RTA underwent CECT of the abdomen using the SB protocol. The axial CECT image shows a massive parenchymal laceration extending to the splenic hilum (black star), with active contrast extravasation (green arrow) confined within the splenic capsule, consistent with the AAST grade IV splenic injury. An incidental note was made of diffusely reduced attenuation of the liver parenchyma (white arrow), suggestive of fatty infiltration, which led to reduced enhancement. AAST: American Association for the Surgery of Trauma; CECT: contrast-enhanced computed tomography; SB: split-bolus; RTA: road traffic accident.

**Figure 4:**
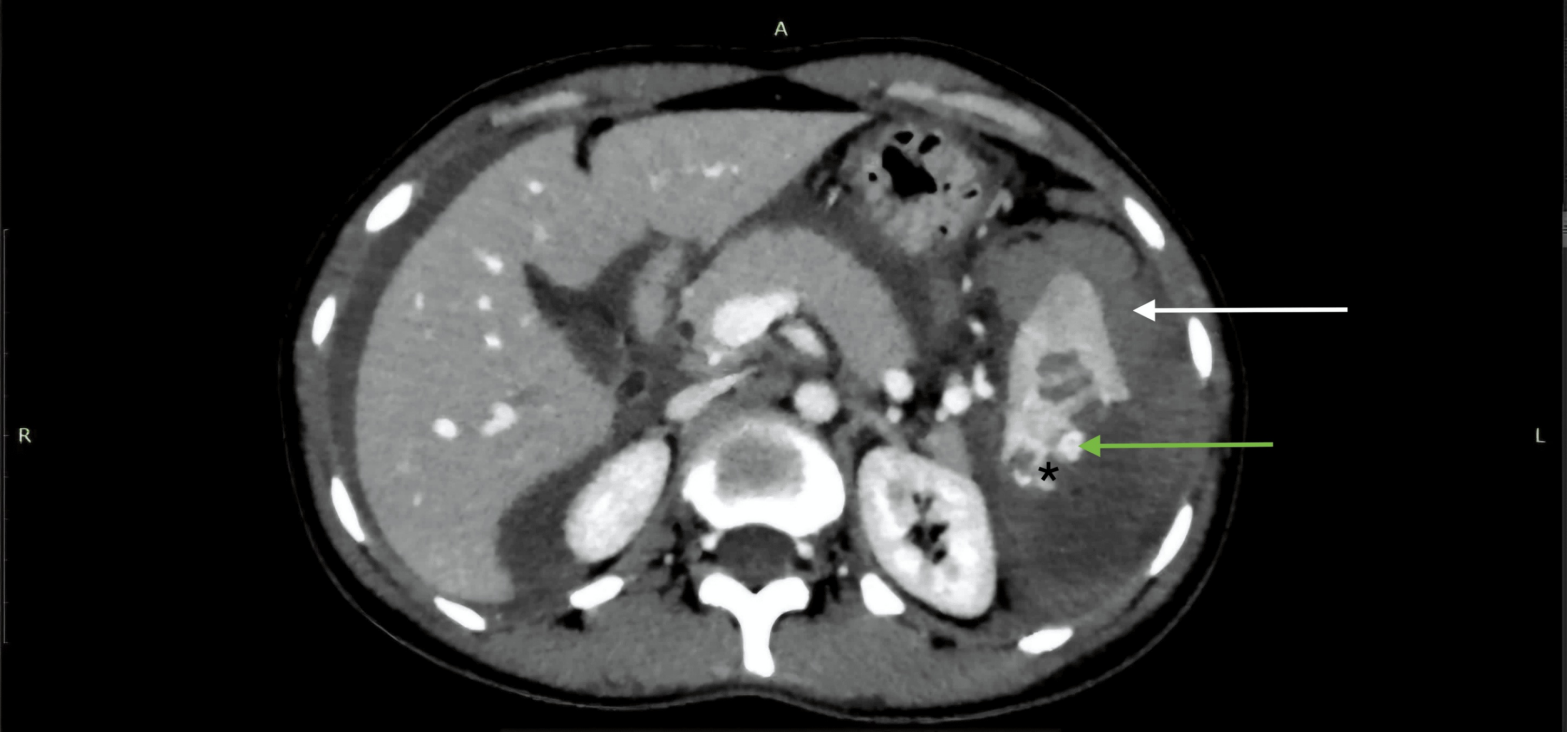
AAST grade IV splenic injury with parenchymal laceration, devascularization, and subcapsular hematoma on CECT (PV protocol). A 31-year-old female with a history of RTA underwent CECT of the abdomen using the PV protocol. The axial CECT image shows a massive parenchymal laceration with approximately 40% devascularization of the splenic parenchyma (black star), a subcapsular hematoma (white arrow), and a small focus of active contrast extravasation (green arrow) confined within the splenic capsule. Findings were consistent with the AAST grade IV splenic injury. AAST: American Association for the Surgery of Trauma; CECT: contrast-enhanced computed tomography; PV: portal venous; RTA: road traffic accident.

**Figure 5:**
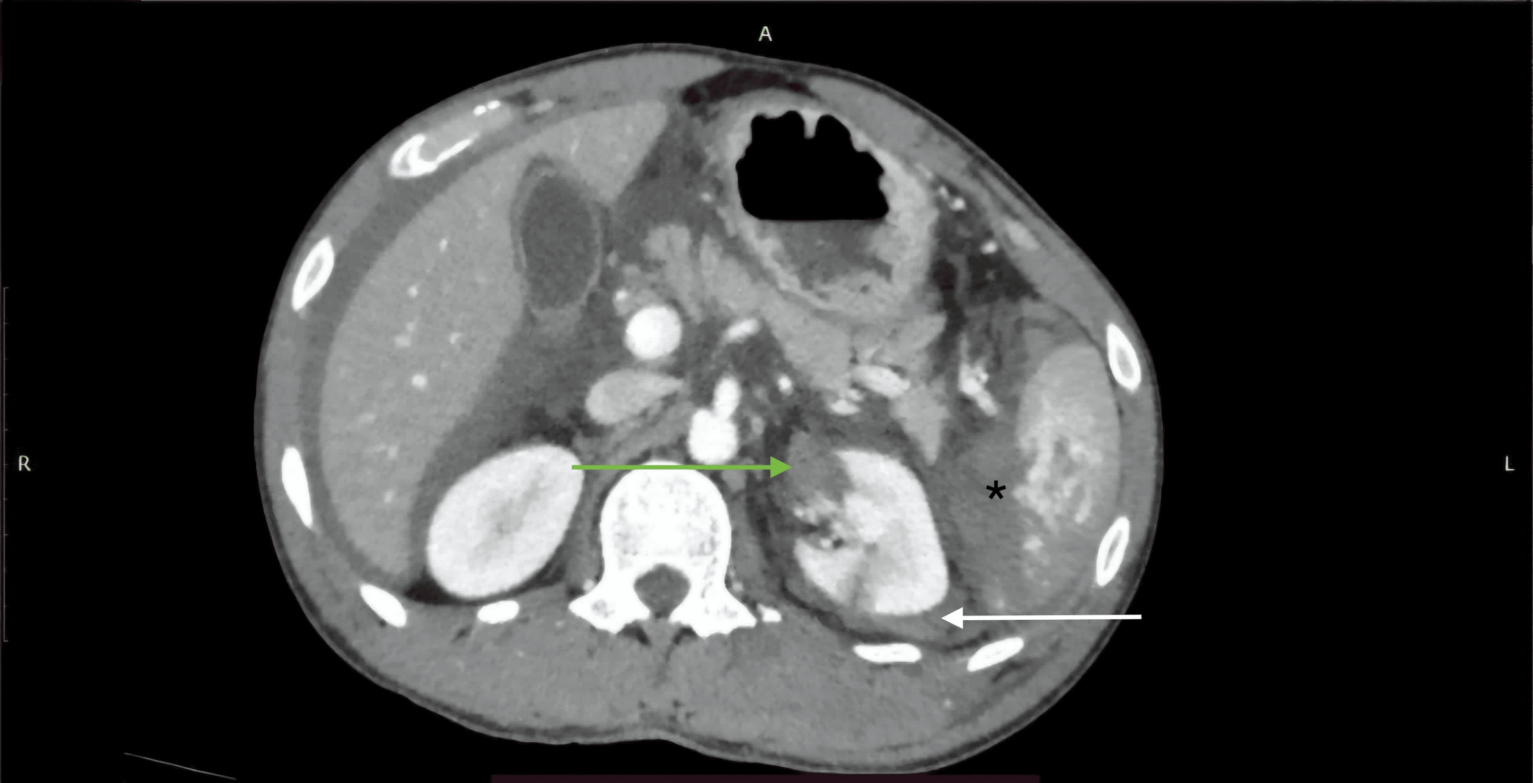
AAST grade III renal injury with wedge-shaped laceration and grade IV splenic injury with devascularization on CECT (SB protocol). A 32-year-old male with a history of RTA underwent CECT of the abdomen using the SB protocol. The axial CECT image reveals a 1.2 cm wedge-shaped laceration affecting the upper pole of the left kidney (green arrow), accompanied by a perirenal hematoma (white arrow). Additionally, a large splenic parenchymal laceration extending up to the hilum with approximately 40% devascularization of the splenic parenchyma (black star) and moderate hemoperitoneum is noted. Findings were consistent with the AAST grade III renal injury and grade IV splenic injury. AAST: American Association for the Surgery of Trauma; CECT: contrast-enhanced computed tomography; SB: split-bolus; RTA: road traffic accident.

**Figure 6:**
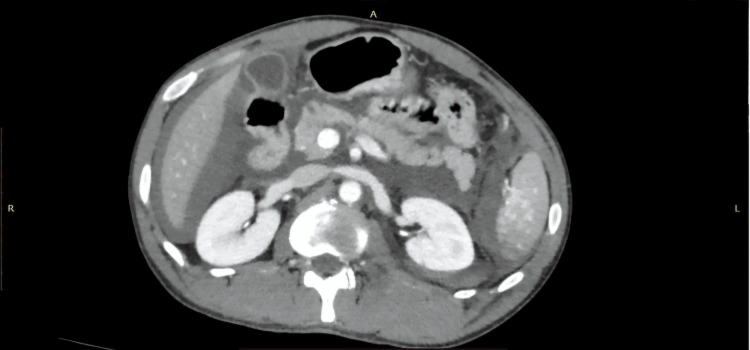
Simultaneous bilateral renal PCS opacification without extravasation on CECT (SB protocol). In the same patient (Figure 5), the use of the SB protocol resulted in simultaneous opacification of the bilateral renal pelvicalyceal system without any contrast extravasation. As a result, a delayed scan was not required, thereby reducing additional radiation exposure to the patient. PCS: pelvicalyceal system; CECT: contrast-enhanced computed tomography; SB: split-bolus.

**Figure 7:**
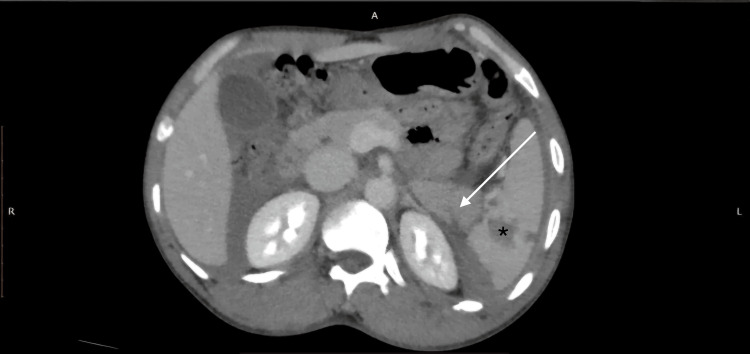
AAST grade I pancreatic injury with superficial laceration and grade III splenic injury with subcapsular hematoma on CECT (PV protocol). A 35-year-old male with a history of alcohol intoxication under unknown circumstances underwent CECT of the abdomen using the PV protocol. The axial CECT image shows a small superficial pancreatic laceration (white arrow) without ductal injury. Additionally, the spleen exhibits an intraparenchymal laceration (black star) extending to the splenic hilum, with a subcapsular hematoma but no active contrast extravasation. Moderate hemoperitoneum was also noted. Findings were consistent with the AAST grade I pancreatic injury and grade III splenic injury. AAST: American Association for the Surgery of Trauma; CECT: contrast-enhanced computed tomography; PV: portal venous.

Liver injuries were most commonly grade II in 18 (25.7%) and grade III in 13 (18.6%) patients, followed by grade I in four (5.7%) and grade IV in two (2.9%), with no grade V injuries observed (Table 3 and Figures 1, 2). Splenic injuries were distributed across all grades, except grade V, with grade II in seven (10.0%), grade I in six (8.6%), grade III in six (8.6%), and grade IV in five (7.1%) patients (Table 3 and Figures 3, 4). Kidney injuries were predominantly grade II in three (4.3%) and grade III in three (4.3%) cases, with one grade IV injury (1.4%), and no grade I or grade V injuries were noted (Table 3 and Figures 5, 6). Pancreatic injury was limited to a single grade I case (1.4%) (Table 3 and Figure 7).

**Table 3:** Distribution of intra-abdominal solid organ injuries by the Organ Injury Scale (OIS) grade, expressed as n (%). The table summarizes the grading of solid organ injuries in patients with blunt abdominal trauma. The liver was the most frequently injured organ (n = 37), followed by the spleen (n = 24), kidney (n = 7), and pancreas (n = 1). Percentages are calculated based on the total study population (N = 70).

Solid organ injury	Grade I	Grade II	Grade III	Grade IV	Grade V
Liver (n = 37)	4 (5.7%)	18 (25.7%)	13 (18.6%)	2 (2.9%)	0 (0%)
Spleen (n = 24)	6 (8.6%)	7 (10.0%)	6 (8.6%)	5 (7.1%)	0 (0%)
Kidney (n = 7)	0 (0%)	3 (4.3%)	3 (4.3%)	1 (1.4%)	0 (0%)
Pancreas (n = 1)	1 (1.4%)	0 (0%)	0 (0%)	0 (0%)	0 (0%)

Comparison of the SB and PV protocols demonstrated significant differences in contrast enhancement across multiple abdominal organs and vessels. The mean image quality scores (five-point Likert scale) were 4.2 ± 0.6 for SB and 4.1 ± 0.7 for PV (p = 0.47), indicating no significant difference in perceived image quality between the two protocols. Splenic injuries were detected in 8/35 patients (22.9%) in the SB group and 16/35 patients (45.7%) in the PV group (p = 0.044). However, the SB protocol provided superior enhancement in several structures. The mean splenic enhancement was higher with SB (141.7 ± 27.8 HU) than PV (119.6 ± 19.8 HU, p = 0.001). Similarly, pancreatic enhancement was greater in SB (121.7 ± 22.1 HU) compared to PV (99.9 ± 13.6 HU, p = 0.001). The right renal cortex showed significantly higher attenuation with SB (240.6 ± 45.1 HU) versus PV (179.9 ± 31.4 HU, p = 0.001). Renal pelvicalyceal system (PCS) enhancement was markedly improved with SB (754.5 ± 334.6 HU) compared to PV (46.4 ± 6.6 HU, p = 0.001). Vascular enhancement was also consistently higher in the SB group, including the portal vein (212.7 ± 58.4 HU vs. 168.1 ± 31.7 HU, p = 0.001), suprarenal IVC (153.4 ± 37.1 HU vs. 127.3 ± 26.1 HU, p = 0.001), suprarenal aorta (319.3 ± 69.3 HU vs. 163.7 ± 32.6 HU, p = 0.001), infrarenal IVC (139.1 ± 40.9 HU vs. 119.9 ± 27.0 HU, p = 0.022), infrarenal aorta (318.5 ± 69.8 HU vs. 169.4 ± 34.6 HU, p = 0.001), right common iliac artery (279.6 ± 84.6 HU vs. 160.3 ± 37.8 HU, p = 0.001), and right common iliac vein (167.4 ± 80.3 HU vs. 123.8 ± 30.4 HU, p = 0.018). These findings indicate that the SB protocol achieved consistently superior vascular and parenchymal enhancement while maintaining comparable hepatic visualization and radiation exposure between the two groups (Table 4).

**Table 4:** Statistically significant differences in organ and vascular enhancement between split-bolus (SB) and portal venous (PV) protocols. Comparison of spleen injury frequency and organ/vascular enhancement values between split-bolus (SB) and portal venous (PV) CT protocols. Values are presented as mean ± standard deviation or percentage. The χ² test was used for categorical variables, and Welch’s t-test was applied for continuous variables. PCS: pelvicalyceal system; IVC: inferior vena cava; HU: Hounsfield units.

Parameter	SB protocol (mean ± SD)	PV protocol (mean ± SD)	p-value	Test statistics
Spleen injury (%)	8 (22.9%)	16 (45.7%)	0.044	χ2 = 4.06
Spleen enhancement (HU)	141.7 ± 27.8	119.6 ± 19.8	0.001	t = 3.83
Pancreas enhancement (HU)	121.7 ± 22.1	99.9 ± 13.6	0.001	t = 4.97
Right kidney cortex (HU)	240.6 ± 45.1	179.9 ± 31.4	0.001	t = 6.53
Renal PCS enhancement (HU)	754.5 ± 334.6	46.4 ± 6.6	0.001	t = 12.52
Portal vein enhancement (HU)	212.7 ± 58.4	168.1 ± 31.7	0.001	t = 3.97
Suprarenal IVC (HU)	153.4 ± 37.1	127.3 ± 26.1	0.001	t = 3.40
Suprarenal aorta (HU)	319.3 ± 69.3	163.7 ± 32.6	0.001	t = 12.02
Infrarenal IVC (HU)	139.1 ± 40.9	119.9 ± 27.0	0.022	t = 2.32
Infrarenal aorta (HU)	318.5 ± 69.8	169.4 ± 34.6	0.001	t = 11.32
Right common iliac artery (HU)	279.6 ± 84.6	160.3 ± 37.8	0.001	t = 7.62
Right common iliac vein (HU)	167.4 ± 80.3	123.8 ± 30.4	0.018	t = 3.00

The agreement between reader 1 and reader 2 in the OIS scoring was moderate for the SB protocol (weighted kappa = 0.563, p = 0.003) and substantial for the PV protocol (weighted kappa = 0.639, p < 0.001). Overall agreement between the two readers was 75.0% in the SB group and 76.5% in the PV group, corresponding to moderate-to-substantial inter-reader reliability, consistent with the weighted kappa statistics.

The SB protocol demonstrated significantly higher enhancement in most abdominal organs and vascular structures compared with PV. The greatest enhancement was noted in the spleen, renal cortex, renal PCS, portal vein, and major iliac and aortic vessels (p < 0.001 for all). The largest effect sizes were observed in the renal PCS (0.84), suprarenal aorta (0.82), and infrarenal aorta (0.81), reflecting a strong association between protocol type and contrast enhancement.

Radiation exposure was also similar between the protocols, with a mean dose of 8.87 ± 2.85 mSv in the SB group and 8.89 ± 3.05 mSv in the PV group. Median radiation doses were 8.1 mSv and 8.5 mSv, respectively, with ranges of 5.1-17.7 mSv in SB and 4.7-16.1 mSv in PV, confirming that both protocols offered equivalent radiation exposure (Table 2).

## Discussion

This study compared the SB and routine PV CT protocols in patients with blunt abdominal trauma to evaluate whether SB could serve as a viable alternative. Over one year, 70 patients were enrolled, with 35 in each group. Males comprised the majority (75.7%), while females accounted for 24.3%. The most frequently affected age group was 31-40 years (31.4%), followed by 21-30 years (22.9%) and ≤20 years (14.3%). These findings are consistent with those reported by Ghosh et al. [16], who noted a higher incidence of blunt abdominal trauma in young males, likely due to greater exposure to high-risk activities.

Among the study participants, 11.4% had no intra-abdominal solid organ injury despite FAST positivity, most often presenting with musculoskeletal injuries. The liver was the most commonly injured organ (52.9%), followed by the spleen (34.3%), adrenal glands (15.7%), kidneys (10.0%), and pancreas (1.4%). Liver injuries were most often grade II (18 cases) and grade III (13 cases), with fewer cases of grade I (four cases) and grade IV (two cases), while no grade V injuries were seen. Splenic injuries were distributed across all grades, except grade V, most commonly grade II (seven cases), grade III (six cases), grade I (six cases), and grade IV (five cases). Kidney injuries were predominantly grade II (three cases) and grade III (three cases), with a single grade IV case. Pancreatic injury was rare, limited to a single grade I case. These findings are comparable to those of Arumugam et al., who also reported the liver as the most frequently affected solid organ in blunt trauma [17]. Conversely, other studies from regions with high rates of chronic infection and splenomegaly have identified the spleen as more vulnerable to rupture, explaining the variability in reported patterns [18]. Adrenal injuries, though relatively uncommon in most trauma series, appeared at a higher frequency in our cohort than renal injuries. This may be attributable to the small sample size, referral bias, or the improved sensitivity of modern MDCT in detecting subtle adrenal hemorrhages, and therefore should be interpreted with caution.

Both protocols demonstrated similar liver enhancement (SB: 106.57 HU; PV: 105.51 HU, p = 0.834), indicating equivalent performance in hepatic imaging. In contrast, SB provided significantly superior enhancement in the spleen (141.71 HU vs. 119.60 HU, p < 0.001, r = 0.42), pancreas (121.69 HU vs. 99.97 HU, p < 0.001, r = 0.51), and right renal cortex (240.57 HU vs. 179.94 HU, p < 0.001, r = 0.62). Psoas muscle enhancement showed no significant difference between SB and PV (62.74 HU vs. 60.09 HU, p = 0.069). Overall, SB provided better parenchymal enhancement for most abdominal organs. Radiologists also rated SB images higher on OIS scoring, except for the liver and psoas muscle, where enhancement remained comparable. Reduced liver attenuation was occasionally observed in patients with hepatic steatosis, which may have influenced hepatic enhancement values.

Interestingly, splenic injuries were detected more frequently in the PV group than in the SB group, despite higher mean splenic enhancement with SB. This paradox may be attributed to differences in parenchymal opacification timing, reduced conspicuity of lacerations against a more intensely enhanced splenic background, or reader variability. Since patients were randomly allocated, the higher detection rate in the PV group likely reflects a diagnostic limitation of SB rather than a true difference in injury prevalence. The simultaneous opacification of arterial and venous phases in SB may obscure subtle lacerations, lowering its sensitivity for certain splenic injuries. Therefore, while SB provides superior vascular and parenchymal enhancement overall, dedicated PV imaging may retain an advantage in detecting subtle splenic trauma, and this limitation should be considered in clinical interpretation.

Our results are in agreement with Godt et al. [19], who reported that SB achieved superior contrast enhancement, comparable image quality, and equal diagnostic accuracy for organ injury detection. Beenen et al. [20] similarly concluded that SB offered superior vascular enhancement and image quality compared with other single-pass protocols, though single-volume contrast protocols were faster at the expense of clarity. Additional study by Loupatatzis et al. [21] has also demonstrated the advantages of SB in achieving improved vascular and parenchymal imaging while minimizing unnecessary radiation or excessive image load.

**Figure 8:**
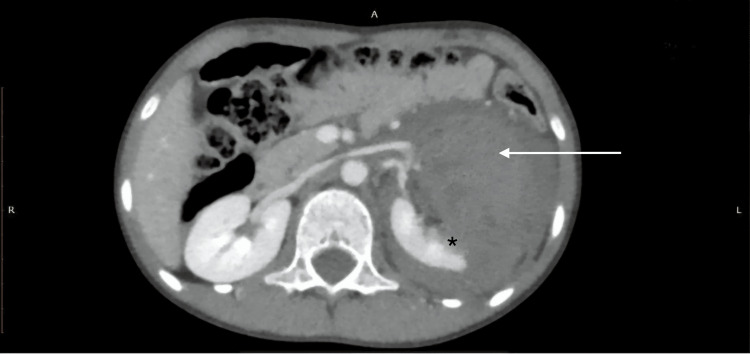
AAST grade IV left renal injury with mid-pole laceration and large perinephric hematoma on CECT (PV protocol). A nine-year-old male child with a history of RTA underwent CECT of the abdomen using the PV phase. The axial CECT image shows a left renal laceration predominantly involving the mid-pole (black star), extending to the renal hilum, with an associated large perinephric hematoma (white arrow). AAST: American Association for the Surgery of Trauma; CECT: contrast-enhanced computed tomography; PV: portal venous; RTA: road traffic accident.

**Figure 9:**
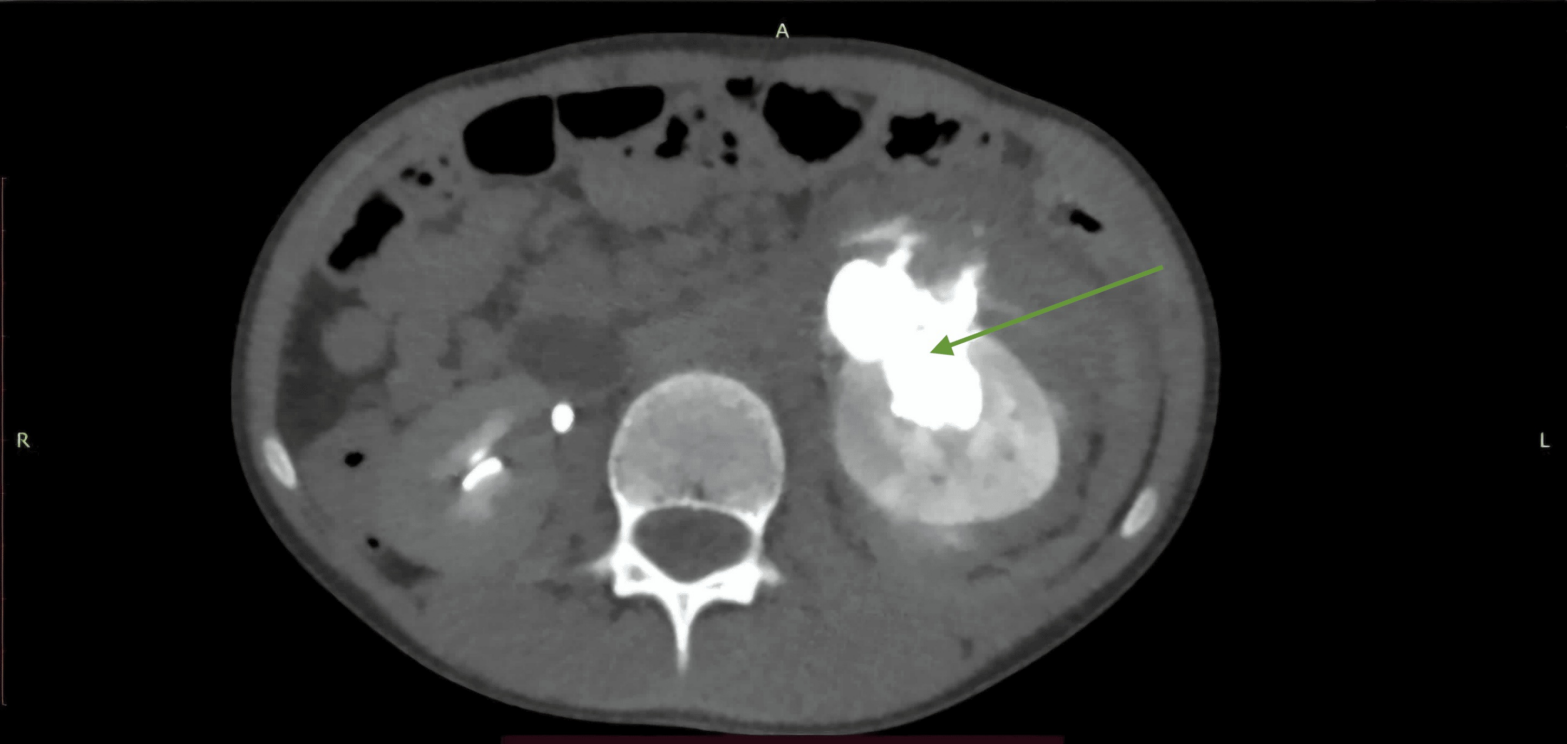
AAST grade IV renal injury with collecting system laceration and contrast extravasation on delayed CECT. A delayed scan in the same patient reveals contrast extravasation (black arrow) from the renal pelvis, indicating a laceration of the collecting system. Findings are consistent with the AAST grade IV renal injury. AAST: American Association for the Surgery of Trauma; CECT: contrast-enhanced computed tomography.

The SB protocol provided significantly higher vascular enhancement across several structures, including the renal PCS, portal vein, suprarenal IVC, suprarenal aorta, infrarenal IVC, infrarenal aorta, right common iliac artery, and right common iliac vein. Importantly, the increased renal PCS enhancement achieved with SB improved urinary tract visualization, thereby reducing the requirement for delayed-phase scans in suspected injuries (Figures 8, 9). However, one limitation was the difficulty in distinguishing renal vascular injuries from collecting system injuries, since both structures opacified simultaneously. Despite this challenge, Beenen et al. [20] reported that arterial injuries could still be accurately identified based on the anatomical location and pattern of contrast extravasation. This improved vascular enhancement in SB may allow more reliable detection of subtle injuries that could be missed with the PV protocol.

The mean CTDIvol and DLP were comparable between SB and PV groups (CTDIvol: X vs. Y mGy; DLP: X vs. Y mGy·cm, both p > 0.05). The derived effective dose was also similar (8.87 ± 2.85 mSv vs. 8.89 ± 3.05 mSv, p = 0.846). This observation aligns with the findings of Leung et al. [22], who demonstrated that SB reduced excess radiation compared with conventional multiphasic trauma imaging protocols. Given the well-established risks associated with ionizing radiation, Mathews et al. [23] emphasized that CT in blunt abdominal trauma is a clearly justified indication, given the high risk of morbidity, and diagnostic accuracy must remain the foremost priority. Nevertheless, protocols should continue to be optimized to minimize unnecessary radiation while preserving diagnostic yield.

Both SB and PV protocols demonstrated comparable diagnostic performance in organ injury assessment, with moderate to substantial inter-reader agreement. Agreement between the two radiologists was 75.0% for SB and 76.5% for PV, with statistically significant concordance. These results are consistent with the observations of Nellensteijn et al. [24], who also reported moderate inter-observer reliability in grading hepatic injuries on CT.

This study has certain limitations. Being a single-center study with a relatively small sample size, the findings may not be generalizable to larger or more diverse trauma populations. Our cohort was enriched with patients at higher risk of intra-abdominal injury due to inclusion criteria requiring a positive FAST or strong clinical suspicion in hemodynamically stable individuals. This likely contributed to the relatively high prevalence of solid organ injuries observed and should be considered when comparing results with broader trauma cohorts. Further multicenter research with larger and more heterogeneous populations is needed to validate these findings and refine the clinical utility of SB.

Another limitation was the use of a non-probability convenience sampling method rather than true randomization, which may have introduced selection bias. Additionally, exclusion of patients with technical issues, such as contrast extravasation and motion-related artifacts, common in real-world trauma workflows, may have limited the generalizability of our results. Future studies should aim to include such cases to better reflect the practical challenges of CT imaging in emergency settings.

A small number of patients were included based on strong clinical suspicion despite a negative FAST result. Sensitivity analysis excluding this subgroup yielded results consistent with the main findings, suggesting their inclusion did not bias outcomes. Another limitation was the relatively high contrast volume (150 mL) compared with many contemporary trauma protocols (80-100 mL). While this ensured robust enhancement across both vascular and parenchymal structures, it may limit generalizability to centers where lower contrast doses are standard. Future investigations should evaluate whether reduced volumes can achieve comparable diagnostic performance in SB protocols.

The Injury Severity Score (ISS) could not be reported, which may limit comparability with other trauma cohorts, and baseline demographic data were not analyzed separately for the SB and PV groups, restricting assessment of group comparability. Finally, while SB provides simultaneous arterial and venous phase opacification, its sensitivity for subtle splenic lacerations and precise differentiation of arterial versus venous injuries may be lower than multiphasic CT, which remains the gold standard for vascular injury localization. SB should therefore be interpreted with this limitation in mind, although its practical utility remains strong in resource-limited settings.

Despite these limitations, the study has several notable strengths. It employed a prospective design, used standardized CT acquisition protocols, and involved blinded dual-reader assessments by experienced radiologists, which minimized bias and improved reliability. Importantly, it provides one of the few direct head-to-head comparisons of SB and PV protocols in blunt abdominal trauma, offering real-world evidence of diagnostic performance.

Overall, the study highlights the potential of SB to enhance vascular and parenchymal visualization without increasing radiation burden. By consolidating arterial and venous phases into a single acquisition, SB reduces the need for additional delayed scans and may improve efficiency in selected settings, even if it does not necessarily simplify workflow compared with multiphasic protocols. With its diagnostic efficiency, radiation safety, and applicability in diverse clinical contexts, SB represents a promising alternative to the conventional PV protocol, though further refinement and validation are warranted before widespread adoption.

## Conclusions

This study demonstrated that the SB CT protocol provides superior parenchymal and vascular enhancement compared with the routine PV protocol in patients with blunt abdominal trauma. The SB technique improved image quality, enabled simultaneous arterial and venous phase visualization, and increased the likelihood of detecting sites of contrast extravasation suggestive of active bleeding in a single acquisition, though definitive confirmation still requires multiphasic imaging. Its inherent delayed-phase component also allowed reliable assessment of urinary tract injuries without the need for additional scans. By consolidating multiple contrast phases into a single acquisition, SB reduced scanning time and minimized the risk of excess radiation exposure. Considering its diagnostic accuracy, workflow efficiency, and radiation safety, the SB protocol represents a promising alternative to conventional PV imaging and may serve as a preferred approach in the comprehensive evaluation and management of blunt abdominal trauma.
